# E-commerce and green food consumption of rural residents: implications for sustainable rural food systems

**DOI:** 10.3389/fnut.2025.1685466

**Published:** 2025-11-05

**Authors:** Hailan Qiu, Xueyi Zhang, Hanyun Deng, Xiangqi Wu, Zhihua Wu, Biao Sheng

**Affiliations:** ^1^School of Economics and Management, Jiangxi Agricultural University, Nanchang, China; ^2^Research Center for the Three Rural Issues, Jiangxi Agricultural University, Nanchang, China; ^3^School of Systems Science and Engineering, Sun Yat-sen University, Guangzhou, China

**Keywords:** e-commerce operations, rural e-commerce, rural residents, green food consumption, sustainable consumption

## Abstract

**Introduction:**

E-commerce is a powerful tool for promoting the green food consumption in rural areas. It can effectively break through the shackles of poor information flow, low income and traditional consumption patterns. This study explores the impact of e-commerce operations on green food consumption of rural residents, focusing on its role in enhancing information access, improving income, and upgrading logistics. It provides a new theoretical support for promoting green food consumption and ensuring food safety.

**Methods:**

Based on survey data from 2,805 rural residents across 10 provinces in China, this study employs a binary Probit model and a mediating effect model to examine the impact and mechanisms of e-commerce operations on green food consumption of rural residents, while also testing for heterogeneous effects. Furthermore, it investigates the differential influences of e-commerce operation models, operation scales, product types, and types of green food.

**Results and discussion:**

The findings indicate that e-commerce operations significantly promote green food consumption of rural residents, and this conclusion remains robust after multiple robustness and endogeneity tests. Mechanism analysis reveals that e-commerce facilitates green food consumption by improving household income, enhancing information acquisition, and strengthening logistics infrastructure. Heterogeneity analysis shows that the positive effect is more pronounced among rural residents with higher social capital, in the central and eastern regions, and in major grain-producing areas. Additional results suggest that platform e-commerce, large-scale operations, and businesses involving primary processed products exert stronger effects on green food consumption, while e-commerce operations more effectively boost the consumption of green vegetables and dairy products. Overall, by examining e-commerce operations, this study provides an in-depth analysis of how online market participation fosters green food consumption of rural residents, offering important insights for advancing sustainable consumption in the context of digital economy transformation.

## 1 Introduction

As China’s ecological civilization construction continues to deepen, promoting the sustainable development of rural consumption is a key and fundamental yet arduous task in exploring the path of rural green development in China (1). The concept of green consumption is gradually extending to rural areas, and green food consumption is becoming a new trend in rural consumption upgrading. However, to further stimulate and release the potential of rural green consumption, it is still necessary to explore key breakthroughs (2). At the same time, the widespread adoption of the Internet and the rapid expansion of e-commerce have brought new opportunities for the transformation of consumption patterns, especially opening up new channels for green food consumption in rural areas (3). Data shows that China’s rural online retail sales reached 2.69 trillion yuan in 2024, laying a solid foundation for rural residents’ online consumption of green food. At the policy level, the “14th Five-Year Plan Implementation Plan for Expanding Domestic Demand Strategy” explicitly emphasizes the pivotal role of e-commerce platforms in cultivating green and low-carbon consumer markets. Through mechanisms such as quality signaling on e-commerce platforms (4) and demand-responsive systems (5), the decision-making framework for rural food consumption is undergoing a structural shift—from price-driven preferences to behaviors oriented toward quality, safety, and ecological sustainability. Meanwhile, some rural residents are deeply embedded in the e-commerce system as business entities (6), and their product cognition, market information, and social resources accumulated in e-commerce practices may further shape their consumption choices. In this context, rural residents, as key players in the coupled system of production and consumption, are increasingly influenced by the e-commerce market. This raises a core question: Does e-commerce operations have a significant impact on their green food consumption behavior? If so, what is the underlying mechanism?

Existing research mainly explored the collaborative mechanism between e-commerce and green consumption. First, e-commerce platforms achieve full-life-cycle traceability of green products through digital technologies, significantly alleviating the problem of information asymmetry (7). Generally, blockchain traceability labels for green agricultural products can increase consumers’ willingness to pay by 23%, as they verify the environmental attributes of products and compliance of production processes through an immutable data chain (4). Similarly, big data algorithms on e-commerce platforms can accurately match farmers’ needs with product information, reduce search costs and make it easier for consumers to find providers of green products, thereby increasing consumption conversion rates (5). In addition, social behaviors play a key role in promoting the popularization of green food (8–10). The interactivity of social media, which features user-generated content (UGC), will further strengthen the trust mechanism for green products. For instance, online buyer reviews on e-commerce platforms can generate a chain mediating effect through intentions linked to subjective norms, thereby stimulating consumption behavior (11). Social e-commerce accelerates the diffusion of green concepts through the demonstration effect of KOLs (Key Opinion Leaders) (12). Frequent exposure to public media helps individuals to form green food consumption intentions during interactions (13–15). Secondly, e-commerce platforms subtly guide green consumption through “nudge” strategies. Studies have shown that visually highlighting environmental labels can increase farmers’ selection probability by 18% (16). Mechanisms such as “green points” or “carbon accounts” on e-commerce platforms (e.g., Ant Forest) further enhance the sustainability of consumption behavior through the dual drivers of material incentives (17). Finally, the synergistic effect between government policies and e-commerce platforms provides institutional guarantee for green consumption. The high efficiency of product supply chains can be achieved through the government-harmonized platform franchising system and the government’s green subsidies system (18). The combination of subsidies and discounts on e-commerce platforms can reduce the premium of green products (19). The public investment in cold chain logistics infrastructure have reduced the circulation loss rate of green food. Especially in remote areas, the improvement of logistics efficiency has increased the availability of green food by 40% (20).

In summary, existing studies have provided valuable insights into e-commerce and green consumption. However, several gaps remain that warrant further investigation. First, most research has focused on the green food consumption behavior of urban residents, while the rural population—despite its growing importance—has been largely overlooked. Second, under the backdrop of rural economic digital transformation, there is a lack of analytical frameworks that explore rural green food consumption from the perspective of e-commerce operations. Third, the influence of e-commerce on green food consumption may be context-dependent, requiring deeper analysis across different dimensions. To address these gaps, this study draws on survey data from 2,805 rural households across 10 Chinese provinces to examine the effects and mechanisms of e-commerce operations on green food consumption of rural residents. It further investigates heterogeneity in the impact across various operation scales, operation models, product types, and green food categories. The findings aim to provide empirical evidence and policy guidance to support the synergistic advancement of digitalization and green transformation in rural areas.

## 2 Theoretical framework and research hypotheses

### 2.1 The direct impact of e-commerce operations on green food consumption of rural residents

Rural e-commerce operates on the basis of grass-roots service networks, such as county service centers and village pick-up points (21, 22), there is a significant difference between its service model and the way urban residents directly interface with mainstream e-commerce platforms, and this scenario specificity determines that the path for rural residents to access e-commerce is very different from that of urban residents. Rural residents are both producers of agricultural products and consumers of food, and under the competition mechanism and consumer feedback system of rural e-commerce, consumers’ concern for food safety, organic certification, traceability and other green attributes will directly motivate rural residents to take the initiative to improve production methods (23), compared with the single role of urban residents as consumers, the green production practices of rural residents are more closely related to their own consumption behaviors, for example, by reducing the use of chemical fertilizers and pesticides and adopting ecological planting techniques to improve the green quality of agricultural products (24, 25), this process will also synchronize and strengthen their own awareness of green food consumption.

According to the theory of cognitive coordination, when rural residents adopt green practices on the supply side, their consumption behavior tends to be more in line with production behavior, thus forming a virtuous cycle of production-consumption, which is difficult to be seen in urban residents due to the lack of participation on the production side (48). It is also one of the core logics of this study focusing on rural residents. In addition, rural residents previously relied on offline markets to buy food, with low accessibility and weak awareness of green products, while e-commerce platforms are more critical to the spread of green food consumption concepts in rural areas (26): on the one hand, rural residents can be exposed to green food consumption trends in the urban high-end market through platform data analysis and consumer evaluation, such as organic food and low-carbon packaging, realizing consumption cognition from scratch; on the other hand, the product comparison and selection opportunities provided by the e-commerce platform can help them intuitively understand the value of green products, gradually get rid of traditional consumption habits, and shift to a healthy, environmentally friendly, quality-oriented consumption model. This change in consumer perception will directly promote rural residents to choose green and sustainable products in food consumption, and ultimately realize the greening and upgrading of the consumption structure, a driving process that is irreplaceable in the rural scene.

Based on the above analysis, this study proposes hypothesis 1:

*H*1: E-commerce operations have a significantly positive impact on green food consumption of rural residents.

### 2.2 Income improvement mechanism

E-commerce operations enhance rural residents’ purchasing capacity by increasing their income levels, thereby promoting their green food consumption. Under traditional agricultural distribution systems, rural producers typically capture only the producer-end price. In contrast, e-commerce platforms reduce intermediary layers and optimize the agricultural value chain, enabling farmers to connect directly with end consumers (20). This shift allows rural residents to access prices closer to retail levels, thereby improving their market position, reducing transaction costs, enhancing counter-cyclical resilience, and minimizing the risk of unsold inventory—all of which contribute to higher household incomes (27). Moreover, in conventional channels, agricultural products are often sold as low-value primary goods. E-commerce platforms, however, provide a showcase for specialty products such as geographical indication goods and organic produce, allowing producers to build brand equity and command price premiums. This value enhancement is not only reflected in immediate transaction prices but also contributes to the development of green brand assets, offering rural households a more stable income growth path. Additionally, the rise of rural e-commerce has stimulated diversification in local economic activities. Beyond direct product sales, the e-commerce ecosystem has spawned complementary industries including food processing, packaging design, and logistics services, offering rural residents expanded employment and entrepreneurship opportunities (28). This diversification significantly improves rural households’ resilience to risk and enables them to move beyond subsistence-oriented consumption toward more aspirational, development-driven consumption of green products. As income increases, budget constraints are relaxed, and rural residents demonstrate stronger demand for high-quality green food.

Based on the above analysis, this study proposes hypothesis 2:

*H*2: E-commerce operations promote green food consumption of rural residents by improving their income levels.

### 2.3 Information access mechanism

E-commerce operations enhance rural residents’ acceptance of green food consumption by improving their access to information, thereby promoting the green food consumption. First, e-commerce platforms continuously push green food-related information, reshaping rural residents’ cognitive framework regarding green products and strengthening their awareness of green food consumption. As a vital channel for information dissemination, e-commerce platforms leverage digital technology to significantly improve the efficiency of spreading information about green food. The multimedia display capabilities of platforms visually showcase the production processes, environmental benefits, and health advantages of green foods, while algorithmic recommendation systems ensure the accuracy and relevance of the information (29). Consumer evaluation systems on e-commerce platforms provide consumers with a rich source of third-party information that profoundly influences their consumption decisions (30–32). Rural residents gradually establish a systematic understanding of these products, laying the knowledge foundation necessary for fostering green food consumption awareness. Second, the interactive features of e-commerce platforms provide innovative pathways for building trust in green food products. The consumer review systems on these platforms serve as rich third-party information sources for rural residents. By reviewing feedback from other consumers, rural residents gain insight into the actual performance and market reputation of green foods. These authentic consumer reviews become an essential basis for evaluating the quality of green food, thus enhancing trust and motivating rural residents to convert their consumption intentions into actual purchases (33). Finally, the information integration features of e-commerce platforms reshape rural residents’ decision-making processes. The unified product information display interface allows easy comparison of key attributes such as quality certifications, pricing structures, and after-sales services across different green food brands. Rural residents can use the platform’s filtering and comparison tools to systematically evaluate products based on personal preferences and budget constraints. This decision-support system not only improves the rationality of consumption choices but also lowers the costs of information search, giving green foods a competitive edge over traditional products and driving a shift toward green consumption patterns.

Based on the above analysis, this study proposes hypothesis 3:

*H*3: E-commerce operations promote green food consumption of rural residents by enhancing their access to information.

### 2.4 Logistics improvement mechanism

**Figure 1:**
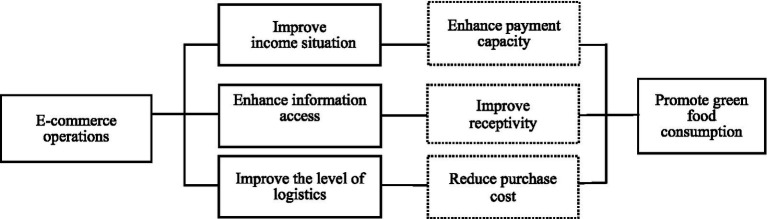
The influence mechanism of e-commerce operations on green food consumption of rural residents.

E-commerce operations reduces the cost of circulation and access to green food by improving the level of logistics services, thus promoting the green food consumption of rural residents. The construction of an efficient and inclusive logistics system is a key link in the promotion of green food consumption by e-commerce, and the essence of this process is the economy of scale effect generated by e-commerce industry agglomeration, and increased investment in logistics infrastructure, such as the construction of modern logistics centers, intelligent warehousing systems, and green transportation networks, can effectively solve the logistical dilemmas, enhance the efficiency of logistical time, and reduce the logistics costs (34). Although the pervasive improvement in end-to-end logistics infrastructure brought about by e-commerce development benefits all rural residents, the intrinsic economic impetus for its construction and maintenance stems primarily from the sustained, scaled demand for orders created by e-commerce operations. It is the e-commerce activities that make the village logistics nodes from “no” to “have,” from “have” to “excellent,” realizing the coverage and upgrading of service functions. The coverage and upgrading of the service function has been realized. The promotion mechanism of green food consumption by improving logistics level is reflected in two aspects: on the one hand, perfect end-to-end logistics significantly reduces the search cost, time cost and transportation loss in the process of green food transaction (35), shortens the waiting time for consumption, and improves the accessibility and freshness of fresh green products, which directly strengthens the purchasing willingness of rural residents. On the other hand, a perfect logistics infrastructure reduces energy consumption and emissions in the logistics process, improves the reliability and satisfaction of logistics services (36, 37), and provides an important guarantee for green food consumption. Consumers pay more attention to the convenience and environmental friendliness of logistics services when choosing green products. While the e-commerce platform responds quickly to market demand, the efficient logistics service promotes the rapid circulation and popularization of green products, improves the green food purchasing experience of rural residents, and strengthens rural residents’ confidence and preference for green food consumption (Figure 1).

Based on the above analysis, this study proposes hypothesis 4:

*H*4: E-commerce operations promote green food consumption of rural residents by improving logistics.

## 3 Methodology

### 3.1 Data sources

The data used in this study are drawn from the 2020 China Rural Revitalization Survey (CRRS), a nationally representative dataset organized by the Rural Development Institute of the Chinese Academy of Social Sciences. The CRRS is a comprehensive survey that covers multiple aspects of rural development, including agricultural production, rural infrastructure, household livelihoods, and social well-being. The survey adopts a combination of stratified and random sampling methods to ensure data representativeness. First, considering economic development levels, geographical locations, and agricultural characteristics, 10 provinces were randomly selected from the eastern, central, western, and northeastern regions of China: Guangdong, Zhejiang, Shandong, Anhui, Henan, Guizhou, Sichuan, Shaanxi, Ningxia, and Heilongjiang. Second, based on per capita GDP, all counties (or equivalent administrative units) within each province were grouped into five strata. One county was then randomly selected from each stratum, yielding five counties per province. Third, within each selected county, three townships were randomly chosen. In each township, villages were categorized into “better-off” and “less-developed” groups, from which one village per group was randomly selected. Finally, using systematic sampling, 12 to 14 rural households were selected from each village roster provided by the local village committee. Trained enumerators conducted face-to-face interviews using structured questionnaires. After excluding observations with missing or abnormal values, a total of 2,805 valid rural household samples were retained for analysis. The focus of this study is on examining the relationship between e-commerce operations and green food consumption of rural residents.

### 3.2 Variable selection

#### 3.2.1 Dependent variable

Following Zhang et al. (38), this study measures green food consumption of rural residents using a binary indicator based on whether the respondent has purchased green food products. The variable is coded as 1 if the respondent has made such purchases, and 0 otherwise.

#### 3.2.2 Core independent variable

This study focuses on assessing the impact of e-commerce operations on green food consumption of rural residents. Drawing on the work of Qiu et al. (39) and considering data availability, e-commerce operations are measured by whether the respondent is engaged in online product sales. The variable is coded as 1 if the household engages in online sales, and 0 otherwise.

#### 3.2.3 Mediating variables

Based on the theoretical framework, e-commerce operations may influence green food consumption through three mediating pathways: income improvement, enhanced information access, and upgraded logistics. Following Qiu et al. (40), this study uses the total household income of rural residents to measure income level. Information access is captured by the respondent’s self-assessed ability to obtain information that meets their needs via the internet. Logistics infrastructure is measured by whether the respondent’s village has direct-to-door parcel delivery service or a designated parcel collection point.

#### 3.2.4 Control variables

In addition to e-commerce operations, other factors may also influence green food consumption of rural residents. Following the approach of Migheli (41), this study incorporates control variables across three dimensions: individual characteristics, household characteristics, and village characteristics. Individual characteristics include gender, age, marital status, education level, political affiliation, risk perception, and self-rated health status. Household characteristics include whether any household member participates in a cooperative and whether there are children under the age of 14 in the household. Village characteristics include village topography, village-level economic conditions, and transportation conditions. Descriptive statistics for all variables are presented in Table 1.

**Table 1:** Definition of variables and descriptive statistical results.

Variables type	Variables name	Definition & coding	Mean	Std. Dev.	Min	Max
Dependent variable	Green Food Consumption	Whether rural residents purchase green food (Yes = 1, No = 0)	0.405	0.491	0	1
Core independent variable	E-commerce Operations	Whether rural residents engage in online product sales (Yes = 1, No = 0)	0.071	0.257	0	1
Mediating variables	Income Status	Total household income (log-transformed, in RMB)	10.824	1.043	7.089	13.941
	Information Access	Can rural residents obtain information that meets their own needs through the internet (Completely dissatisfied = 1, Somewhat dissatisfied = 2, Average = 3, Basically satisfied = 4, Completely satisfied = 5)	4.004	1.035	1	5
	Logistics Level	Whether express delivery reaches the village or is available at a pickup point (Yes = 1, No = 0)	0.261	0.439	0	1
Control variables	Gender	Male = 1, Female = 0	0.955	0.207	0	1
	Age	Age of respondent (years)	54.181	10.355	22	89
	Marital Status	Married = 1, Unmarried = 0	0.985	0.120	0	1
	Education Level	Primary and below = 0, Junior high school and above = 1	0.053	0.225	0	1
	Political Affiliation	Member of CPC = 1, Otherwise = 0	0.244	0.430	0	1
	Risk Perception	Perceived safety of market food (very safe = 1, relatively safe = 2, average = 3, relatively unsafe = 4, very unsafe = 5)	2.848	1.072	1	5
	Health Status	Very poor = 1, relatively poor = 2, average = 3, relatively good = 4, very good = 5	3.613	0.988	1	5
	Cooperative Participation	Any family member in a cooperative (Yes = 1, No = 0)	0.257	0.437	0	1
	Number of Children	Presence of children under 14 in the household (Yes = 1, No = 0)	0.429	0.495	0	1
	Village Topography	Plain = 1, Non-plain = 0	0.446	0.497	0	1
	Village Economic Condition	Per capita income in the village (log-transformed, in RMB)	9.455	0.550	8.132	12.486
	Village Transportation Access	Distance from village committee to county government (km)	23.184	16.693	1	125

See Table 1 for the definition, assignment and statistical characteristics of the above variables.

### 3.3 Model construction

#### 3.3.1 Benchmark regression model

Given that the dependent variable is binary, this study employs a binary Probit model to estimate the effect of e-commerce operations on green food consumption:


(1) $$Y_{i}=\alpha_{0}+\alpha_{1}{\mathrm{Ec}}_{i}+\alpha_{2}X_{i}+\varepsilon_{i}.$$

In Equation (1), Y_i_ denotes green food consumption of rural residents, and Ec_i_ represents e-commerce operations. X_i_ stands for the vector of control variables. α_1_ and α_2_ are the coefficients to be estimated; α_0_ is the constant term; and ε_i_ is the error term.

#### 3.3.2 Mediation effect model

To further examine the mechanisms through which e-commerce operations influence rural green food consumption, the mediation analysis framework proposed by Baron and Kenny (42) is applied. The following system of equations is used:


(2) $$Y_{i}=\alpha_{0}+\alpha_{1}{\mathrm{Ec}}_{i}+\alpha_{2}X_{i}+\varepsilon_{i}.$$

(3) $$Z_{i}=\delta_{0}+\delta_{1}{\mathrm{Ec}}_{i}+\delta_{2}X_{i}+\lambda_{i}.$$

(4) $$Y_{i}=\beta_{0}+\beta_{1}{\mathrm{Ec}}_{i}+\beta_{2}Z_{i}+\beta_{3}X_{i}+\mu_{i}.$$

In Equation (2)–(4), the variables Z_i_ represent the mediators, including income status, information access, and logistics level. α_1_, α_2_, δ_1_, δ_2_, ᵝ_1_, ᵝ_2_, ᵝ_3_ denote the coefficients to be estimated; α_0_, δ_0_, ᵝ_0_ are the constant terms; and ε_i_, $\lambda_{i}$ , μ_i_ are the error terms. All other variables are defined as in Equation (1).

## 4 Results and analysis

### 4.1 Benchmark regression results

The regression results of the impact of e-commerce operations on green food consumption of rural residents are presented in Table 2. Columns (1), (2), and (3) show the regression results after adding individual characteristic variables, household characteristic variables, and village characteristic variables, respectively. The results indicate that e-commerce operations significantly positively affect green food consumption of rural residents, validating Research Hypothesis 1.

**Table 2:** Benchmark regression results.

Variables name	Green food consumption		
	(1)	(2)	(3)
E-commerce operations	0.504***	0.477***	0.441***
	(0.097)	(0.097)	(0.098)
Gender	−0100	−0.101	−0.031
	(0.117)	(0.117)	(0.119)
Age	−0.016***	−0.014***	−0.017***
	(0.002)	(0.003)	(0.003)
Marital status	0.104	0.060	0.064
	(0.208)	(0.210)	(0.214)
Education level	0.226***	0.217***	0.170***
	(0.054)	(0.054)	(0.056)
Political affiliation	0.258***	0.245***	0.235***
	(0.058)	(0.059)	(0.059)
Risk perception	0.006	0.019	0.005
	(0.023)	(0.023)	(0.023)
Health status	0.130***	0.130***	0.125***
	(0.025)	(0.025)	(0.026)
Cooperative participation		0.194***	0.190***
		(0.056)	(0.057)
Number of children		0.114**	0.117**
		(0.051)	(0.051)
Village topography			0.032
			(0.052)
Village economic condition			0.343***
			(0.047)
Village transportation access			−0.004**
			(0.002)
Constant	−0.148	−0.272	−3.293***
	(0.287)	(0.289)	(0.536)
Pseudo R^2^	0.047	0.051	0.071
N	2,805	2,805	2,805

*** and ** mean significant at the statistical level of 1, 5% respectively, and the figures in brackets are standard errors.

Among the control variables, age, education level, political affiliation, health status, cooperative participation, number of children, village economic conditions, and village transportation conditions all exert significant effects on green food consumption of rural residents.

Specifically, among individual characteristics, age shows a significant negative association with green food consumption, suggesting that as age increases, households become less inclined to purchase green food. This may be attributed to older individuals’ lower awareness of green food and their stronger attachment to traditional consumption habits. In contrast, education level and health status are positively and significantly associated with green food consumption. Rural residents with higher levels of education and better health tend to have a clearer understanding of the benefits of green food and a stronger willingness to consume it. Furthermore, political affiliation (i.e., being a member of the Communist Party) is positively associated with green food consumption, likely because party members have greater access to social resources and are more attuned to public interest topics such as environmental protection and health, leading to stronger preferences for green products.

Regarding household characteristics, both the number of children and cooperative participation show significant positive effects. Households with young children tend to place greater emphasis on nutrition and health, which enhances their interest and knowledge of green food. Participation in cooperatives helps rural households expand their social networks and access information and services related to green food, thereby facilitating green food consumption.

As for village-level factors, transportation conditions are negatively associated with green food consumption. Rural residents in remote areas often face limited access to green food products due to poor infrastructure, which hinders their ability to purchase such goods. In contrast, village economic conditions have a significant positive effect. Villages with higher per capita income and greater market development foster stronger green food consumption awareness and purchasing power among rural residents, making them more likely to buy green food products.

### 4.2 Robustness test

#### 4.2.1 Replace the core independent variable

To test the robustness of the above results, this paper uses “the number of operated online stores” as a measurement indicator for e-commerce operations and conducts a regression analysis again. The results are shown in column (1) of Table 3. The findings indicate that e-commerce operations have a significant positive impact on green food consumption of rural residents, and the above conclusion remains valid.

**Table 3:** Robustness test results.

Variables name	(1)	(2)	(3)	(4)	(5)
E-commerce operations	0.301**	0.440***	0.719***	0.364***	0.427***
	(0.142)	(0.098)	(0.001)	(0.111)	(0.098)
Gender	−0.020	−0.058	−0.056	0.032	−0.025
	(0.119)	(0.119)	(0.195)	(0.136)	(0.120)
Age	−0.017***	−0.017***	−0.028***	−0.012***	−0.017***
	(0.003)	(0.003)	(0.004)	(0.004)	(0.002)
Marital status	0.069	0.037	0.106	0.046	0.077
	(0.213)	(0.214)	(0.348)	(0.231)	(0.214)
Education level	0.176***	0.169***	0.279***	0.152**	0.173***
	(0.056)	(0.056)	(0.092)	(0.067)	(0.056)
Political affiliation	0.242***	0.224***	0.382***	0.241***	0.230***
	(0.059)	(0.059)	(0.097)	(0.071)	(0.059)
Risk perception	0.008	−0.003	0.009	−0.026	0.006
	(0.023)	(0.023)	(0.038)	(0.027)	(0.023)
Health status	0.124***	0.119***	0.206***	0.136***	0.125***
	(0.026)	(0.026)	(0.042)	(0.030)	(0.026)
Cooperative participation	0.204***	0.178***	0.312***	0.190***	0.189***
	(0.056)	(0.057)	(0.092)	(0.065)	(0.057)
Number of children	0.120**	0.127**	0.186**	0.077	0.120**
	(0.051)	(0.051)	(0.084)	(0.060)	(0.051)
Village topography	0.029	0.052	0.051	0.050	0.019
	(0.052)	(0.052)	(0.085)	(0.060)	(0.052)
Village economic condition	0.349***	0.358***	0.565***	0.413***	0.409***
	(0.047)	(0.047)	(0.078)	(0.055)	(0.051)
Village transportation access	−0.004***	−0.004***	−0.006***	−0.003**	−0.004***
	(0.002)	(0.002)	(0.003)	(0.002)	(0.002)
Constant	−3.347***	−3.413***	−5.427***	−4.153***	−3.926***
	(0.535)	(0.537)	(0.886)	(0.626)	(0.571)
Pseudo R^2^	0.067	0.071	0.071	0.067	0.074
N	2,805	2,805	2,805	2055	2,805

*** and ** mean significant at the statistical level of 1, 5%, respectively.

#### 4.2.2 Replace the dependent variable

In addition to modifying the key independent variable, this study also redefines the dependent variable by using the quantity of green food consumed by rural households instead of a binary indicator. As shown in Column (2) of Table 3, the regression results remain consistent. E-commerce operations continue to have a significantly positive effect on rural green food consumption, further confirming the robustness of the main findings.

#### 4.2.3 Replace the regression model

Considering that the green food consumption of rural residents is a binary variable, this paper adopts the Logit model instead of the Probit model for re-estimation. The results are shown in column (3) of Table 3, where e-commerce operations are still significantly positive at the 1% statistical level, indicating the robustness of the above regression results.

#### 4.2.4 Replace the sample

To eliminate potential bias introduced by non-labor force respondents who may differ in physical ability or cognitive capacity, individuals under the age of 16 and over 60 who are not in the labor force are excluded from the sample. The results, reported in Column (4) of Table 3, continue to show a significantly positive effect of e-commerce operations on green food consumption, thereby reinforcing the robustness of the earlier findings.

#### 4.2.5 Winsorization

In addition to the above robustness checks, this study also conducts a 1% winsorization of all continuous control variables to mitigate the influence of potential outliers. The regression results, presented in Column (5) of Table 3, show that e-commerce operations continue to have a significantly positive impact on green food consumption of rural residents. This finding is consistent with the baseline results and further supports the robustness of the main conclusions.

#### 4.2.6 Placebo test

**Figure 2:**
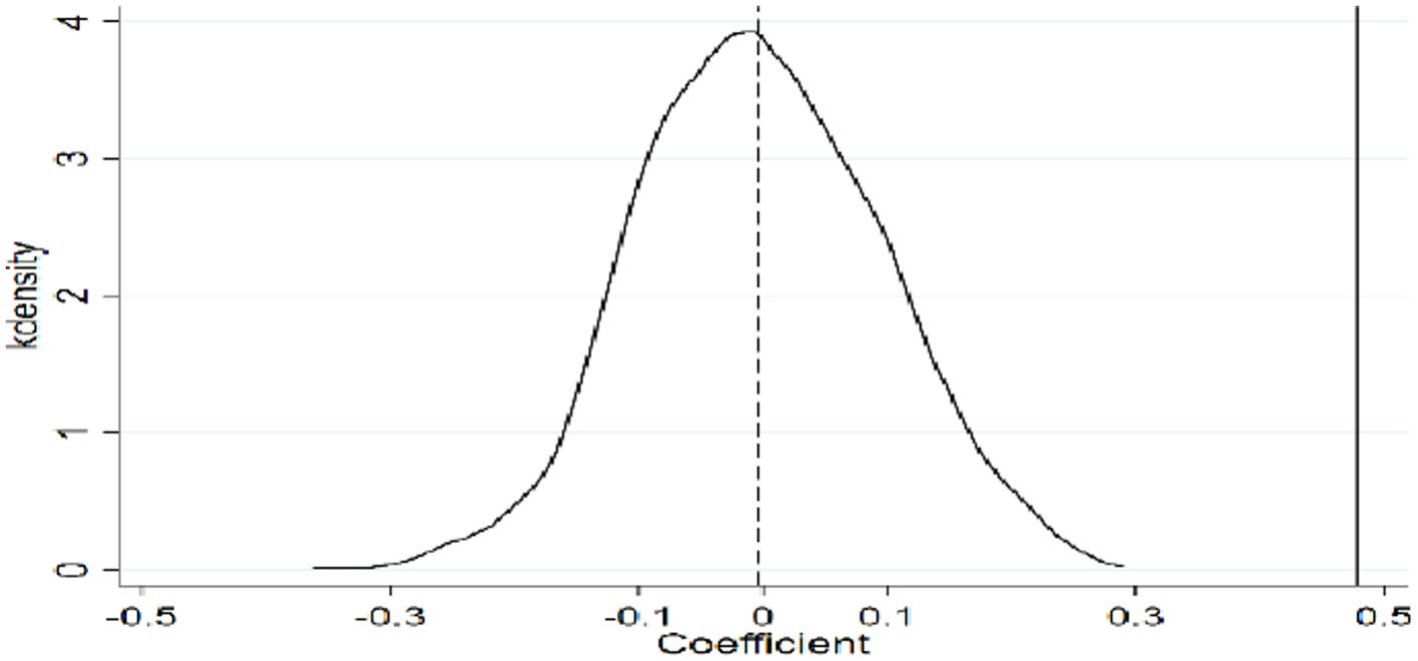
Placebo test.

To further validate the positive effect of e-commerce operations on green food consumption of rural residents and control for potential unobserved or random factors, a placebo test is conducted. The results are shown in Figure 2. A “false” core independent variable is randomly assigned to the treatment and control groups, and the baseline model is re-estimated. After repeating the randomization process 1,000 times, the results show that the mean of the simulated kernel density distribution is concentrated around zero, while the true regression coefficient is significantly distant from this distribution. This confirms that the observed effect of e-commerce operations on green food consumption is not driven by unobserved factors, reinforcing the robustness of the baseline regression results.

### 4.3 Endogenous test

Given that e-commerce operations are self-selected behaviors among rural residents, the previous model may suffer from endogeneity due to potential self-selection bias. To address this issue, the Propensity Score Matching (PSM) method is employed. The PSM regression results in Table 4 show that regardless of whether propensity score matching methods such as nearest neighbor matching, radius matching, caliper matching, kernel matching, local linear regression matching, or Mahalanobis matching are used for estimation, e-commerce operations have a significant positive impact on green food consumption of rural residents. These results reinforce the reliability of the baseline findings by accounting for potential selection bias.

**Table 4:** PSM estimation results.

Matching method	Treated group	Control group	ATT	Std. error	T-value
Nearest neighbor (1-to-1)	0.621	0.363	0.258***	0.052	4.91
K-nearest neighbors	0.621	0.449	0.172***	0.042	4.13
Radius matching	0.621	0.388	0.233***	0.036	6.41
Caliper matching (1-to-4)	0.621	0.449	0.172***	0.042	4.13
Kernel matching	0.621	0.438	0.183***	0.037	4.98
Local linear regression matching	0.621	0.460	0.161***	0.052	3.06
Mahalanobis matching	0.621	0.416	0.205***	0.052	3.91

*** means significant at the statistical level of 1%.

**Figure 3:**
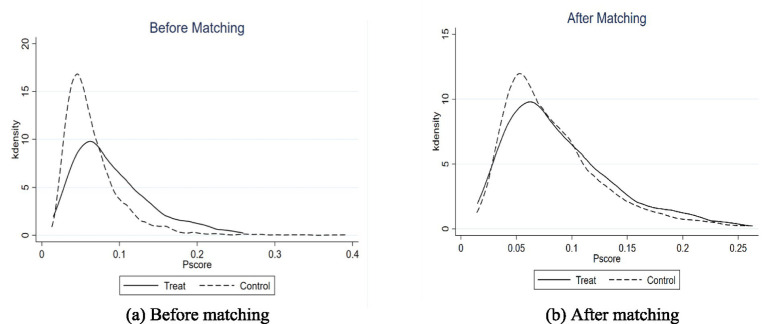
Distribution of propensity scores before and after matching.

The PSM model specification must satisfy two prerequisite conditions: the overlap assumption and the balance characteristic (43). For the overlapping assumption, the common support domain needs to be tested. Figure 3 shows the distribution of propensity scores for testing the common support domain, where a is distribution of propensity scores before matching and b is distribution of propensity scores after matching. After matching, the e-commerce operations samples and non-e-commerce operations samples almost overlapped in the propensity score, and there was a large common support interval, which indicates that the matching was relatively reasonable.

To evaluate whether the propensity score matching (PSM) effectively balances the data, a covariate balance test was conducted. The results are presented in Table 5. Taking the nearest neighbor matching method (1-to-1) as an example, the findings indicate that, compared to the pre-matching sample, the standardized mean differences for most control variables were substantially reduced after matching, with all falling below the commonly accepted threshold of 10%. Furthermore, the t-test results for these covariates fail to reject the null hypothesis of no systematic differences between the treatment and control groups. This suggests that the distribution of covariates between the two groups was successfully balanced. Therefore, the PSM results pass the balance test, confirming the robustness of the matching procedure.

**Table 5:** Covariate balance test results.

Variables name	Pre-matching mean		Post-matching mean		Bias (%)		T-test	
	Treatment	Control	Treatment	Control	Before	After	t-Statistic	*p* > t
Gender	0.953	0.955	0.953	0.953	−1.3	0.0	−0.00	1.000
Age	51.595	54.367	51.595	52.179	−26.6	−5.6	−0.52	0.601
Marital status	0.990	0.985	0.990	0.989	3.9	0.0	0.00	1.000
Education level	0.768	0.651	0.768	0.774	26.0	−1.2	−0.12	0.903
Political affiliation	0.342	0.237	0.342	0.416	23.4	−16.3	−1.48	0.139
Risk perception	2.911	2.843	2.911	2.932	6.3	−2.0	−0.19	0.847
Health status	3.695	3.605	3.695	3.668	9.3	2.7	0.26	0.792
Cooperative participation	0.379	0.247	0.379	0.379	28.6	0.0	0.00	1.000
Number of children	0.500	0.422	0.500	0.505	15.5	−1.1	−0.10	0.919
Village topography	0.426	0.447	0.426	0.373	−4.2	10.6	1.05	0.296
Village economic condition	9.598	9.446	9.598	9.537	27.6	11.0	0.97	0.331
Village transportation access	23.828	23.151	23.828	24.258	4.0	−2.5	−0.24	0.807

### 4.4 Mechanism analysis

To further investigate the mechanisms through which e-commerce operations influences green food consumption of rural residents, this study conducts a mediation analysis. The regression results are reported in Table 6. Columns (1), (2), and (3) reveal that e-commerce operations significantly improves household income, which in turn promotes green food consumption of rural residents. This finding provides empirical support for Hypothesis 2. Similarly, by examining Columns (1), (4), and (5), it is evident that e-commerce operations enhances information access, which subsequently facilitates green food consumption. These results validate Hypothesis 3. Lastly, the results from Columns (1), (6), and (7) indicate that e-commerce operations improve the level of logistics services, thereby encouraging rural residents to increase their green food consumption. This provides empirical evidence supporting Hypothesis 4.

**Table 6:** Mechanism analysis results.

Variables name	(1)	(2)	(3)	(4)	(5)	(6)	(7)
	Green food consumption	Income status	Green food consumption	Information access	Green food consumption	Logistics level	Green food consumption
E-commerce operations	0.441***	0.459***	0.387***	0.137**	0.435***	0.260**	0.584***
	(0.098)	(0.073)	(0.099)	(0.076)	(0.098)	(0.102)	(0.098)
Income status			0.128***				
			(0.026)				
Information access					0.091***		
					(0.026)		
Logistics level							0.278***
							(0.058)
Control variables	Controlled	Controlled	Controlled	Controlled	Controlled	Controlled	Controlled
Constant	−3.293***	7.089***	−4.223***	3.181***	−3.597***	−3.238***	−3.107***
	(0.536)	(0.390)	(0.571)	(0.403)	(0.544)	(0.613)	(0.570)
Pseudo R^2^/ R^2^	0.071	0.160	0.078	0.084	0.075	0.220	0.072
N	2,805	2,805	2,805	2,805	2,805	2,805	2,805

*** and ** mean significant at the statistical level of 1, 5%, respectively.

### 4.5 Heterogeneity test

#### 4.5.1 Level of social capital

Social capital, encompassing the trust, norms, and cooperative relationships that rural residents accumulate through their social networks, can significantly influence their efficiency in resource acquisition, ability to identify credible information, and the effectiveness of value transmission during e-commerce activities. Consequently, the impact of e-commerce operations on green food consumption may vary depending on the level of social capital. To explore this heterogeneity, this study examines how the effect of e-commerce operations on rural green food consumption differs across groups with varying levels of social capital. The results are presented in Table 7. The findings indicate that e-commerce operations has a stronger positive effect on green food consumption of rural residents with high levels of social capital compared to those with low levels of social capital. A plausible explanation is that individuals in high social capital groups tend to possess broader social networks and more robust access to information, making it easier for them to obtain knowledge about green food. Moreover, their behavior is more likely to be influenced by social identity and peer recognition, thereby increasing the likelihood of engaging in green food consumption.

**Table 7:** Heterogeneity analysis by level of social capital.

Variables name	Green food consumption	
	Low social capital	High social capital
E-commerce operations	0.404***	0.434***
	(0.133)	(0.148)
Control variables	Controlled	Controlled
Constant	−3.052***	−2.260**
	(0.644)	(1.158)
Pseudo R^2^	0.061	0.078
N	1885	920

*** and ** mean significant at the statistical level of 1, 5%, respectively.

#### 4.5.2 Regional distribution

Significant disparities exist across regions in terms of resource endowments, institutional environments, and levels of economic development. These differences can influence rural residents’ awareness of and capacity for green food consumption, thereby affecting the impact of e-commerce operations on green food consumption outcomes. To examine this heterogeneity, the sample provinces were grouped into two categories: the central and eastern regions, and the western region. A subgroup regression analysis was then conducted, and the results are reported in Table 8. The findings reveal that e-commerce operations have a more pronounced effect on promoting green food consumption of rural residents in the central and eastern regions, compared to those in the western region. This may be attributed to the higher level of economic development and the more mature green food consumption market in the central and eastern regions. These favorable external conditions facilitate the effective conversion of information advantages and channel accessibility—brought about by e-commerce—into tangible consumption behavior among rural residents.

**Table 8:** Heterogeneity analysis by regional distribution.

Variables name	Green food consumption	
	Central and eastern regions	Western region
E-commerce operations	0.443***	0.397**
	(0.126)	(0.163)
Control variables	Controlled	Controlled
Constant	−2.208***	−5.593***
	(0.699)	(0.998)
Pseudo R^2^	0.081	0.102
N	1,607	1,198

*** and ** mean significant at the statistical level of 1, 5%, respectively.

#### 4.5.3 Agricultural functional zones

Agricultural functional zones differ significantly in terms of production structure, resource endowments, and industrial policy frameworks. These differences may influence how e-commerce operations affect green food consumption of rural residents. In this study, the sample provinces were categorized based on whether they belong to major grain-producing areas, thereby forming distinct agricultural functional zones. The heterogeneity of e-commerce’s impact across these zones was then examined. The results are presented in Table 9. The findings suggest that the effect of e-commerce operations on green food consumption is stronger in major grain-producing areas compared to non-grain-producing regions. A possible explanation is that the agricultural infrastructure in major grain-producing regions is more developed, and environmental regulations are more rigorously enforced. As a result, e-commerce activities in these areas are more likely to adhere to green production standards. This enhances rural residents’ trust in green food products, thereby increasing their willingness to engage in food green consumption.

**Table 9:** Heterogeneity analysis by agricultural functional zones.

Variables names	Green food consumption	
	Major grain-producing areas	Non-grain-producing areas
E-commerce operations	0.586***	0.274**
	(0.160)	(0.127)
Control variables	Controlled	Controlled
Constant	−3.823***	−1.877**
	(0.792)	(0. 778)
Pseudo R^2^	0.078	0.089
N	1,407	1,398

*** and ** mean significant at the statistical level of 1, 5%, respectively.

### 4.6 Further analysis

#### 4.6.1 Differences across e-commerce operation models

In rural China, two representative e-commerce operation models for agricultural product sales have emerged over time. The first is the platform e-commerce operation model, in which rural residents operate online stores via large platforms such as Taobao or JD. The second is the social e-commerce model, which relies on social networking tools such as WeChat or Weibo for product promotion and sales. These two models differ significantly in terms of operational logic, resource integration mechanisms, and marketing strategies. To investigate whether these differences lead to varying effects on rural green food consumption, separate regression analyses were conducted for the two models. The results, shown in Table 10, indicate that the platform e-commerce model exerts a greater positive impact on green food consumption of rural residents than the social e-commerce model. A plausible explanation lies in the stricter quality assurance requirements embedded in platform e-commerce operations. Sellers must adhere to product certification procedures and quality control standards, which necessitate a working knowledge of green food labeling systems and product specifications. This continuous exposure enhances sellers’ awareness of the value and credibility of green food. Additionally, platform e-commerce integrates more comprehensive green food supply chain resources, offering sellers better access to high-quality products. Platform algorithms, customer feedback mechanisms, and traffic flows also help channel sellers’ attention toward green consumption markets, reinforcing their own consumption behaviors. In contrast, the social e-commerce operation model relies more heavily on interpersonal networks and informal communication. It places less emphasis on product standardization and professional knowledge of green food, which limits sellers’ exposure to high-quality sources and reduces the likelihood that their operations will influence their own green food consumption behavior.

**Table 10:** Impact differences in e-commerce operation model.

Variables name	Platform E-commerce	Social E-commerce
E-commerce operations	0.481**	0.352***
	(0.286)	(0.108)
Control variables	Controlled	Controlled
Constant	−3.367***	−3.344***
	(0.535)	(0.536)
Pseudo R^2^	0.067	0.069
N	2,805	2,805

*** and ** mean significant at the statistical level of 1, 5%, respectively.

#### 4.6.2 Differences across e-commerce operation scales

Significant differences exist between e-commerce operators of varying operation scales in terms of resource acquisition capabilities and market awareness. To further explore this heterogeneity, the present study examines the impact of e-commerce operations on green food consumption of rural residents under different operation scales. The results are presented in Table 11. The findings indicate that large-scale e-commerce operators have a stronger positive effect on green food consumption of rural residents compared to small-scale or informal e-commerce participants. One possible explanation is that large-scale e-commerce activities require rural operators to engage more deeply with market trends, quality standards, and certification systems related to green food. This level of engagement enhances their understanding of the value and importance of green food, which, in turn, shapes their personal consumption preferences. Additionally, large-scale e-commerce operators typically have access to more extensive and reliable supply chain networks, which provide easier and lower-cost access to diverse and high-quality green food products. Their operational experience also leads to the accumulation of specialized knowledge in areas such as storage and freshness preservation, thereby increasing the convenience and feasibility of personal consumption. Moreover, as promoters of green food products to customers, large-scale e-commerce operators are more likely to serve as behavioral exemplars, reinforcing their own green food consumption habits. Income gains from large-scale further strengthen their financial capacity to purchase green food. In contrast, small-scale e-commerce operators generally face limitations in market insights, resource access, purchasing power, and role-model effects, which constrain the extent to which their business activities influence their own green food consumption behaviors.

**Table 11:** Impact differences in e-commerce operation scales.

Variables name	Small-scale E-commerce	Large-scale E-commerce
E-commerce operations	0.390***	0.440*
	(0.117)	(0.266)
Control variables	Controlled	Controlled
Constant	−3.334***	−3.387***
	(0.536)	(0.535)
Pseudo R^2^	0.069	0.067
N	2,805	2,805

*** and ** mean significant at the statistical level of 1, 5%, respectively.

#### 4.6.3 Difference across e-commerce operation product types

The types of products managed by rural e-commerce operators are closely related to their exposure to resources, market cognition, and operational habits—all of which can indirectly influence their own green food consumption decisions. To examine this dimension of heterogeneity, the present study investigates how different product types in e-commerce operations affect green food consumption of rural residents. The regression results are reported in Table 12. The findings show that operating e-commerce businesses focused on primary processed agricultural products has a stronger positive effect on green food consumption than operating e-commerce businesses dealing in unprocessed agricultural products. A possible explanation lies in the nature of primary processing, which requires the operator’s involvement in product selection, handling, and packaging—processes that typically demand stricter quality control and higher green standards for raw materials. This involvement often leads to increased exposure to professional knowledge such as green certification systems and quality testing procedures. Such deep attention to green attributes may naturally translate into personal consumption decisions. Furthermore, the e-commerce of primary processed products often involves more standardized supply chain management and branding practices. Through interactions with upstream and downstream partners, these operators are more likely to encounter diverse green food sources. Additionally, the added value generated through processing enhances their purchasing power. In contrast, e-commerce operations focused on unprocessed agricultural products are generally limited to the direct sale of raw materials, with less emphasis on quality verification or professional knowledge accumulation. The exposure to green food sources is also more likely to be limited to a single category, thereby reducing the likelihood of influencing the operator’s own green food consumption.

**Table 12:** Impact differences in e-commerce operation product types.

Variables name	Agricultural products E-commerce	Primary processed products E-commerce
E-commerce operations	0.208*	0.327***
	(0.116)	(0.108)
Control variables	Controlled	Controlled
Constant	−3.350***	−3.328
	(0.536)	(0.536)
Pseudo R^2^	0.067	0.068
N	2,805	2,805

*** and ** mean significant at the statistical level of 1, 5%, respectively.

#### 4.6.4 Differences across types of green food

Different types of green food vary significantly in terms of their product attributes, consumption scenarios, price sensitivity, and distribution characteristics. To further examine these distinctions, this study investigates the heterogeneous effects of e-commerce operations on the consumption of different types of green food. The results are presented in Table 13. The findings show that e-commerce operations more significantly promote the consumption of vegetables and dairy products compared to meat products. This result may be explained by the fact that vegetables and dairy products are more compatible with the operational advantages of e-commerce, such as standardized packaging and cold-chain logistics, which help ensure product quality. Moreover, these products typically have lower unit prices and are consumed more frequently in daily life. The broad range of options and convenient purchasing channels accessible through e-commerce platforms can quickly meet the everyday dietary needs of rural residents. In addition, promotional campaigns and traceability features provided by e-commerce platforms further reduce concerns related to food safety and authenticity for these product types. In contrast, meat products—especially fresh meat—pose greater logistical challenges due to higher cold-chain requirements and transport costs. Rural residents also tend to prefer purchasing meat through offline markets where they can visually assess freshness and quality. Given the higher unit price and larger quantity per purchase, the logistical and informational advantages of e-commerce may not fully compensate for these preferences. Furthermore, meat consumption in rural areas is often shaped by habitual dietary patterns, with relatively inelastic demand. As a result, the ability of e-commerce operations to stimulate increased meat consumption is weaker compared to its influence on vegetable and dairy consumption.

**Table 13:** Impact differences in type of green food.

Variables name	Vegetables	Meat	Dairy products
E-commerce operations	0.390***	0.279	0.910***
	(0.170)	(0.243)	(0.265)
Control variables	Controlled	Controlled	Controlled
Constant	−3.029***	−3.341**	−10.126***
	(0.912)	(1.298)	(1.417)
R^2^	0.048	0.041	0.077
N	2,805	2,805	2,805

*** and ** mean significant at the statistical level of 1, 5%, respectively.

## 5 Discussion

Based on 2,805 micro-survey data from 10 provinces of China, this paper empirically analyzes the impact and mechanism of e-commerce operations on green food consumption of rural residents by using binary Probit model and mediation effect model, and examines the heterogeneous impact of e-commerce operations on green food consumption of rural residents. On this basis, the differences in the impact of different e-commerce operations modes, operation scales, operation product types and green food types are further discussed. In order to promote green food consumption, ensuring food safety provides new theoretical support.

Specifically, this study finds that e-commerce operations significantly promote green food consumption of rural residents. Peng et al. (44) also discussed e-commerce and green consumption using Baidu Green Consumption Index of 31 provinces and cities in China from 2016 to 2020, but reached the opposite conclusion from this paper. This may be due to the multiple differences in research object, research level and mechanism focus. First of all, on the research object, the existing research focuses on the broad sense of “green consumption,” which covers energy-saving products, environmental protection materials and other types of goods, while this paper focuses on the specific field of “green food consumption,” which is more directly related to health demands, food safety and rural industrial characteristics. Therefore, the information asymmetry problem of e-commerce platform may be more effectively alleviated in the food category through direct connection of origin and traceability technology. Secondly, in the research group, the existing research is based on the whole population sample, but this paper focuses on the rural residents, whose consumption behavior is more restricted by income constraints, limited access to information and insufficient logistics infrastructure. For rural residents, e-commerce operations is not only a consumption channel, but also an important way to improve income and market access. It plays a role through the triple mechanism of income increase effect, information empowerment and logistics optimization, thus directly activating the potential demand for green food. In addition, at the data level, there are studies based on macro data that may be difficult to capture the micro-mechanism of individual behavior change, while this paper relies on micro-survey data to identify the direct impact and intermediary path of e-commerce operations on rural residents ‘consumption decision-making in more detail.

When discussing the influence mechanism between e-commerce operations and green food consumption of rural residents, this paper finds that e-commerce operations promotes green food consumption of rural residents by improving income status, strengthening information acquisition and upgrading logistics level. Among them, the income effect and information effect of Internet and e-commerce platform have been generally recognized by scholars (45–47). In rural areas, green food consumption has long been limited by the “last mile” obstruction, lack of cold chain and high circulation costs, which makes it difficult to convert consumption intention into actual purchase behavior. By embedding and activating the localized logistics network, improving the storage preservation conditions and distribution efficiency, e-commerce operations directly enhances the accessibility, timeliness and convenience of the purchase process of green food. Therefore, bringing logistics level promotion into intermediary mechanism broadens the traditional perspective of e-commerce and consumption research, and provides a new analytical dimension for understanding how digital technology reshapes rural material circulation and consumption mode.

Therefore, compared with the existing research, this study breaks through the macro narrative of the traditional e-commerce-green consumption relationship, reveals the differential function mechanism of e-commerce operations in rural situation from the micro level, emphasizes the unique response mode of green food consumption as a commodity with high income elasticity, and provides more situational sensitive theoretical explanation and empirical basis for e-commerce to promote rural revitalization and green food consumption transformation. Finally, this paper makes a detailed heterogeneity analysis and further discussion, which provides a more solid theoretical basis and operational policy enlightenment for understanding the activation of rural green food consumption market.

However, there are some limitations in this paper. Firstly, due to the limitation of data availability, this paper uses cross-section data instead of panel data, which makes it impossible to dynamically capture the dynamic effect with time between e-commerce operations and green food consumption behavior. In addition, the paper has not been able to further explore and test the internal linkage relationship between mediation mechanisms and other potential conduction paths. Future research can collect panel data and construct a more comprehensive mechanism test model to further verify the robustness and universality of the conclusions of this paper, so as to build a more comprehensive and dynamic understanding framework.

## 6 Conclusion and policy implications

### 6.1 Research conclusion

Based on the above analysis, this paper draws the following four conclusions.

First, e-commerce operations have significantly promoted the green food consumption of rural residents.

Second, e-commerce operations promote green food consumption of rural residents by improving household income, enhancing information access, and upgrading logistics infrastructure.

Third, the impact of e-commerce on green food consumption exhibits significant heterogeneity across different levels of social capital, geographic regions, and agricultural functional zones. Specifically, e-commerce operations have a stronger positive effect among rural residents with higher levels of social capital, those located in the central and eastern regions, and those residing in major grain-producing areas.

Fourth, platform e-commerce, large-scale e-commerce and primary processed products e-commerce can promote green food consumption of rural residents; compared with meat consumption, e-commerce operations can promote green vegetable and dairy consumption more.

### 6.2 Policy implications

Based on these findings, the study provides the following policy implications:

First, expand rural e-commerce coverage by continuing to invest in internet infrastructure and reducing connectivity costs. Policymakers should establish standardized e-commerce service stations and comprehensive demonstration projects, and improve county-, township-, and village-level logistics networks to effectively address the “last mile” delivery challenge and ensure that e-commerce services reach rural areas.

Second, strengthen the green food consumption effect by providing targeted measures such as tax incentives and credit support to increase rural residents’ income. In terms of information empowerment, focus on digital skills training and the development of green food information-sharing platforms that integrate production data, market prices, and testing results, thereby enhancing rural residents’ ability to identify green food. Regarding logistics optimization, investment in cold chain and other green distribution infrastructure should be increased to reduce circulation costs and ensure product quality and accessibility.

Third, implement differentiated policies tailored to local contexts. For rural residents with higher social capital, promote green food consumption through model households and organizational experience-sharing sessions. In central and eastern regions with favorable geographic and economic conditions, deepen the integration of e-commerce and the green food industry to establish regional green brands. In major grain-producing areas, support the digital transformation of green food enterprises and strengthen certification systems to encourage the development of local green food consumption habits.

Fourth, release the green consumption potential through multi-dimensional strategies. Encourage the development of standardized platform e-commerce and strengthen regulation and support. Provide fiscal incentives for green food consumption and optimize user interfaces for rural consumers to increase engagement. Support the growth of large-scale and primary-processed food e-commerce, improve green standards and technical guidance, and optimize supply chains and marketing strategies, gradually enhancing the compatibility and accessibility of green food on e-commerce platforms, with particular attention to meat products. These measures should work collectively to promote a comprehensive optimization of rural green food consumption patterns.

## Data Availability

The original contributions presented in the study are included in the article/supplementary material, further inquiries can be directed to the corresponding authors.
